# Comparison of DNA extraction methods for COVID-19 host genetics studies

**DOI:** 10.1371/journal.pone.0287551

**Published:** 2023-10-30

**Authors:** Ronaldo Celerino da Silva, Suelen Cristina de Lima, Wendell Palôma Maria dos Santos Reis, Jurandy Júnior Ferraz de Magalhães, Ronaldo Nascimento de Oliveira Magalhães, Brijesh Rathi, Alain Kohl, Marcos André Cavalcanti Bezerra, Lindomar Pena

**Affiliations:** 1 Department of Virology and Experimental Therapy (LAVITE), Aggeu Magalhães Institute (IAM), Oswaldo Cruz Foundation (Fiocruz), Recife, Pernambuco, Brazil; 2 Department of Genetics, Federal University of Pernambuco (UFPE), Recife, Pernambuco, Brazil; 3 Pernambuco State Central Laboratory (LACEN/PE), Serra Talhada, Pernambuco, Brazil; 4 University of Pernambuco (UPE), Serra Talhada Campus, Serra Talhada, Pernambuco, Brazil; 5 Department of Chemistry, Federal Rural University of Pernambuco (UFRPE), Recife, Brazil; 6 Laboratory for Translational Chemistry and Drug Discovery, Department of Chemistry, Hansraj College, University of Delhi, Delhi, India; 7 MRC-University of Glasgow Centre for Virus Research, Glasgow, Scotland, United Kingdom; Imam Abdulrahman Bin Faisal University, SAUDI ARABIA

## Abstract

The coronavirus disease 2019 (COVID-19) pandemic has resulted in global shortages in supplies for diagnostic tests, especially in the developing world. Risk factors for COVID-19 severity include pre-existing comorbidities, older age and male sex, but other variables are likely play a role in disease outcome. There is indeed increasing evidence that supports the role of host genetics in the predisposition to COVID-19 outcomes. The identification of genetic factors associated with the course of SARS-CoV-2 infections relies on DNA extraction methods. This study compared three DNA extraction methods (Chelex^®^100 resin, phenol-chloroform and the QIAamp DNA extraction kit) for COVID-19 host genetic studies using nasopharyngeal samples from patients. The methods were compared regarding number of required steps for execution, sample handling time, quality and quantity of the extracted material and application in genetic studies. The Chelex^®^100 method was found to be cheapest (33 and 13 times cheaper than the commercial kit and phenol-chloroform, respectively), give the highest DNA yield (306 and 69 times higher than the commercial kit and phenol-chloroform, respectively), with the least handling steps while providing adequate DNA quality for downstream applications. Together, our results show that the Chelex^®^100 resin is an inexpensive, safe, simple, fast, and suitable method for DNA extraction of nasopharyngeal samples from COVID-19 patients for genetics studies. This is particularly relevant in developing countries where cost and handling are critical steps in material processing.

## Introduction

The COVID-19 pandemic caused by the severe acute respiratory syndrome coronavirus-2 (SARS-CoV-2) has posed an unprecedented challenge to humanity at many levels. Soon after SARS-CoV-2 emergence, the virus proved to have sustained human to human transmission and in specific settings, also high mortality [1].

Social distancing, mask wearing, hand hygiene and testing were the pillars of COVID-19 control prior to the advent and deployment of effective vaccines. The gold standard for COVID-19 diagnosis is viral detection through the amplification of nucleic acids in nasopharyngeal swab samples by real time polymerase chain reaction (RT-PCR) [2–4]. In addition, other nucleic acid amplification testing (NAAT) has been utilized in COVID-19 diagnosis, as droplet digital PCR (ddPCR), reverse-transcription loop-mediated isothermal amplification (RT-LAMP) and CRISPR-Cas9 [5, 6]. As SARS-CoV-2 spread globally, a shortage in supplies for diagnostic tests and reagents for scientific research became an issue to many countries, especially in the developing world. This has had an impact in the diagnostics of COVID-19 and other diseases [7].

COVID-19 is a complex disease that present a broad spectrum of clinical manifestations ranging from an asymptomatic to a severe clinical course. Severely ill patients often have multiorgan failure, which may be induced by the cytokine storm, caused by an increased level of inflammatory mediators, endothelial dysfunction, coagulation abnormalities and inflammatory cells infiltration. Severe patients may present acute lung failure, acute liver failure, acute kidney injury, cardiovascular disease, a wide spectrum of hematological abnormalities, neurological disorders, and other abnormalities [8].

Several risk factors have been associated with disease severity, including age, male sex and comorbidities. However, other variables such as host genetic background have been implicated in the outcome of this infection [9, 10]. In addition to a well-designed and characterized study, the quality of extracted host DNA underpins any subsequent work.

A good extraction method needs to be safe, fast to perform and generate genomic DNA with good quality and in sufficient quantity for downstream analyses [11–13]. The main DNA extraction techniques routinely used include organic extraction (phenol–chloroform method), nonorganic method (salting out and proteinase K treatment), adsorption-based methods (silica–gel membrane) and magnetic beads-based methods. These techniques allow consistent DNA isolation from several biological specimens, but they differ in both the quality and the quantity of DNA yielded [11, 13, 14].

In-house methods usually have low cost, but they are oftentimes very laborious and time consuming, and requires many steps involving detergent-mediated lysis, proteinase treatment, extractions with hazardous organic solvents (phenol, chloroform and isoamylalcohol) and ethanol precipitation. Taken together, this increases the risk of DNA cross-contamination from sample to sample and poses chemical and ergonomic risks for the operator. Despite the existence of non-toxic extraction procedures, these require extensive dialysis or the use of filters, making their execution extremely laborious [15, 16]. Commercial DNA isolation kits offer shorter extraction times, do not require specific training, have minimum equipment requirements and result in a good DNA quality. However, the price per sample makes the process very expensive, and even prohibitive, for many laboratories in developing countries [17].

Chelex^®^100 resin has emerged as a safe, economic, and sensitive method for nucleic acid extraction from many sample types, without DNA damage [18]. Studies have shown that its efficiency in DNA extraction in several types of samples, including those comprising low numbers of cells [18–23].

The method used for DNA extraction can have a significant impact on host genetic studies [24]. Given that nasopharyngeal samples are one of the most widely sample used for COVID-19 diagnostics, they can also reveal substantial information on host genetic markers involved in the susceptibility and resistance to SARS-CoV-2 infection [25, 26]. The present work compared three DNA extraction methods (Chelex^®^100, Phenol-chloroform and the QIAamp DNA Mini Kit) to isolate genomic DNA from nasopharyngeal swab samples and evaluated their performance and suitability for genotyping studies.

## Material and methods

### Samples

A total of 100 nasopharyngeal swab samples were collected from individuals displaying respiratory symptoms and SARS-CoV-2 diagnosis was confirmed by qRT-PCR. The nasopharyngeal swab samples were collected using a cotton swab and placed in 3 mL virus transport medium, refrigerated, and shipped to the Pernambuco State Central Laboratory of Public Health “*Dr*. *Milton Bezerra Sobral (Lacen-PE)”*, Brazil, for COVID-19 diagnosis. All samples were collected during the first wave of the COVID-19 pandemic in Pernambuco State in 2020. All samples were stored at -70°C until use.

### Ethical approval

This study was approved by the “Universidade Federal de Pernambuco (UFPE)” Institutional Review Board under protocol CAAE: 44390221.6.0000.5208 and was performed in accordance with relevant guidelines e regulations, including the Brazilian National Health Council (CNS) Resolution 466/2012. The requirement for informed consent study was waived because we used spent samples submitted to COVID-19 diagnosis and all patient identifying information was kept confidential.

### DNA extraction

The genomic DNA extraction was performed using three different protocols: Chelex^®^100 resin (Sigma-Aldrich), Phenol-chloroform (Sigma-Aldrich), and QIAamp DNA Mini Kit (Qiagen). A total of 250 microliters of nasopharyngeal swab sample was used for each protocol.

### Extraction protocols

#### Chelex^®^100 resin

A 250 μL of nasopharyngeal swab sample aliquot was added to 100 μL of Chelex^®^100 resin (5g/mL). The mixture was vortexed and incubated at 56°C for 1 hour in a 0.5 mL microtube in a dry bath. Next, it was heated to 96°C for 30 minutes. After heating, the samples were centrifuged at 13000 rpm for 6 minutes. The supernatant was transferred to a clean microtube of 1.5 mL and 100 μL of ultrapure water were added for rehydration. The genomic DNA obtained was stored at 20°C.

#### Phenol-chloroform

A volume of 250 μL of nasopharyngeal swab sample was added to 400 μL of TKM II buffer in a 2 mL tube. Then, 25 μL of 10% SDS and 5 μL of proteinase K were added and well mixed with a sterile tip. The sample was incubated in a dry bath at 55°C for 30 minutes. A 180 μL of 5M NaCl was added, mixed, and incubated at room temperature for 15 minutes. Centrifugation was performed at 13000 rpm for 10 minutes. The DNA containing supernatant was recovered and placed in a sterile tube (2 mL). 400 μL of chloroform/isoamyl alcohol (Sevag) and 400 μL of saturated phenol (pH 7.6–8) were added, then homogenized by vortexing. The proportion between chloroform/isoamyl alcohol and saturated phenol solutions should always be 1/1. Centrifugation was performed at 13000 rpm for 10 minutes. The DNA containing supernatant was transferred to a new sterile tube (2 mL) and 800 μL of chloroform/Isoamyl alcohol was added, vortexed, and centrifuged at 13000 rpm for 10 minutes. The supernatant was collected into a fresh tube where 10% of the supernatant volume (e.g. 80 μL) of 3M sodium acetate (pH 5.2) was added and 800 μL of ice-cold (-20°C) p.a. ethanol was added. The tube was shaken by inversion to precipitate the DNA and centrifuged at 13000 rpm for 10 minutes to aggregate the precipitated DNA on the tube wall. The supernatant was discarded and 500 μL of ice-cold 70% ethanol are added. The tube was mixed until the DNA detached from the tube wall, resuspended in ethanol and centrifuge at 13000 rpm for 7 minutes. The supernatant was discarded, and the DNA was allowed to dry in a dry bath at 60°C with the tube open and resuspended in 50 μL of TE buffer or deionized water.

#### QIAamp DNA mini kit (Qiagen)

A total of 250μL of sample were used for DNA extraction using the QIAamp® DNA Mini and Blood Mini Handbook according to “DNA Purification from Buccal Swabs (Spin Protocol)” from the manufacturer (https://www.qiagen.com/us/resources/resourcedetail?id=62a200d6-faf4-469b-b50f-2b59cf738962&lang=en). In brief, 400 μl PBS were added to the sample, followed by the addition of QIAGEN protease stock solution and buffer AL. The mixture was incubated at 56°C for 10 minutes, washed with 100% ethanol and the provided buffers and then eluted in 150 μL Buffer AE.

### Protocol runtime

To evaluate the average execution time of each protocol using a manageable number of samples (n = 24) to be extracted in a single run, we measured the time spent by three different operators from beginning to end. We chose this sample size because it is the maximum capacity of tubes that can be processed in a regular benchtop centrifuge in a single run in our setting. The operators were equally trained and familiar with each protocol.

### DNA concentration and purity

DNA concentration and quality were assessed by spectrophotometry using a NanoDrop 2000 Spectrophotometer (Thermo Fisher Scientific) following the manufacturer´s instructions. DNA purity was estimated by determing 260/280 and 260/230 ratios.

### DNA integrity on agarose gel

The integrity and presence of genomic DNA extracted by the three methods was analyzed on 1.5% agarose gel. DNA aliquots (3μL) were run at 100 V and 80 Amps for 30 minutes, stained with GelRed (Biotium) and visualized under an ultraviolet transilluminator (Loccus L-PIX).

### DNA amplification

Genomic DNA was amplified by polymerase chain reaction restriction fragment length polymorphism (PCR-RFLP) and quantitative real time polymerase chain reaction (qRT-PCR) targeting the single nucleotide polymorphism (SNP) rs1801133 in *methylene tetrahydrofolate reductase* (*MTHFR*) gene as a representative marker for host genetic studies. The *MTHFR* gene is one of the regulatory enzymes involved in folate metabolism and this SNP has been associated with various types of diseases, including hypertension [27].

For each extraction method, 20 individuals were randomly selected and genotyped by both RFLP and real-time PCR. In the *MTHFR* PCR-RFLP, the forward primer 5-TGAAG GAGAAGGTGTCTGCGG-3 and the reverse primer 5-AGGACGGTGCGGTGAGAGTG-3 were used. DNA amplification was performed using the commercial kit GoTaq G2 Flexi DNA Polymerase (Promega). Reactions were prepared in a final volume of 25μL: 5μL of GoTaq Flexi Buffer, 2.5 μL of 10mM dNTP mix, 1.5 μL of 25mM MgCl_2_, 0.5 μL of forward primer, 0.5 μL of reverse primer, 0.2 μL of GoTaq G2 flexi DNA Polymerase (5u/μL), 12.8 μL ultrapure water and 2 μL of DNA sample. The cycling conditions used were 94°C for 5 minutes, followed by 35 cycles of 94°C for 1 minute, 62°C for 30 seconds, 72°C for 30 seconds and a final extension of 72°C for 7 minutes. The amplification success was verified by amplicon of 198 bp presence in a 1% agarose gel (data not shown).

The amplicons were digested using the restriction enzyme *HinfI* (Thermo Fisher Scientific). The reactions were prepared for a final volume of 25μL, being 16.8μL of ultrapure water, 3 μL Buffer R, 0.3 of *HinfI* enzyme (10 U/μL) and 10μL of amplicon. The reactions were subjected to a temperature of 37°C overnight (minimum of 12 hours), followed by inactivation at 85°C for 5 minutes. The G/A transition creates a restriction site for the *HinfI*, generating fragments of varying sizes: the homoziygote for the wild-type allele (GG) produces a 198 bp fragment; the heterozygote (GA) produces 198, 175 and 23 bp fragments and the mutant homozygote (AA) produces 175 and 23 bp fragments. the analysis of the fragments was performed using a 3% agarose gel staing with GelRed^®^ (Biotium).

For the qRT-PCR, the *MTHFR* rs1801133 SNP was genotyped using allelic specific probes (TaqMan SNP Genotyping Assays C_1202883_20) in an ABI7500 Real Time PCR machine (Applied Biosystems), according to manufacturer’s recommendations. Each reaction was prepared for a final volume of 10μL, being 5μL of TaqMan^TM^ Genotyping Master Mix (Thermo Fisher Scientific, 0.5 μL of TaqMan Genotyping assay (20x), 1.0 μL de genomic DNA (in concentration of 25 ng) and 3,5μL of ultrapure water. In relation to thermically conditions, the reactions were subjected to pre-PCR read (60° for 1 minute), and holding stage (95°C by 10 minutes), following by cycling stage (50 cycles of 95°C for 15 seconds and 60°C for 1 minutes and 30 seconds) and post-PCR (60°C for 1 minute).

### Cost analysis of each DNA extraction method

To analyse the costs of the several consumables used in each DNA extraction method used, the value per one reaction was calculated based on Brazilian reagent prices at the time of the study (first semester of 2022) and were converted to US $.

### Statistical analysis

The graphics and statistical analysis were done in GraphPad Prism 5 version 5.1 for Windows. DNA concentration, yield and purity values were evaluated for normality through the Shapiro-Wilk normality test. If they presented normal distribution, the methods were compared through the analysis of variance (ANOVA) with the post hoc Tukey test. If they presented non-normal distribution, the methods were compared through the Kruskal-Wallis test followed by the Dunn’s Multiple Comparison test. In all the test were considered a significance level of 0.05.

## Results and discussion

DNA extraction is a cornerstone procedure for genetics and molecular biology studies. For best use in developing countries, methods should be fast, practical, affordable, free of contaminants and toxicity, and give DNA of high quantity and quality [13, 17, 28]. We have looked at alternative solutions for DNA extraction for downstream applications and compared three different DNA extraction protocols to choose the best extraction method for nasopharyngeal swab samples of COVID-19 patients.

**Fig 1:**
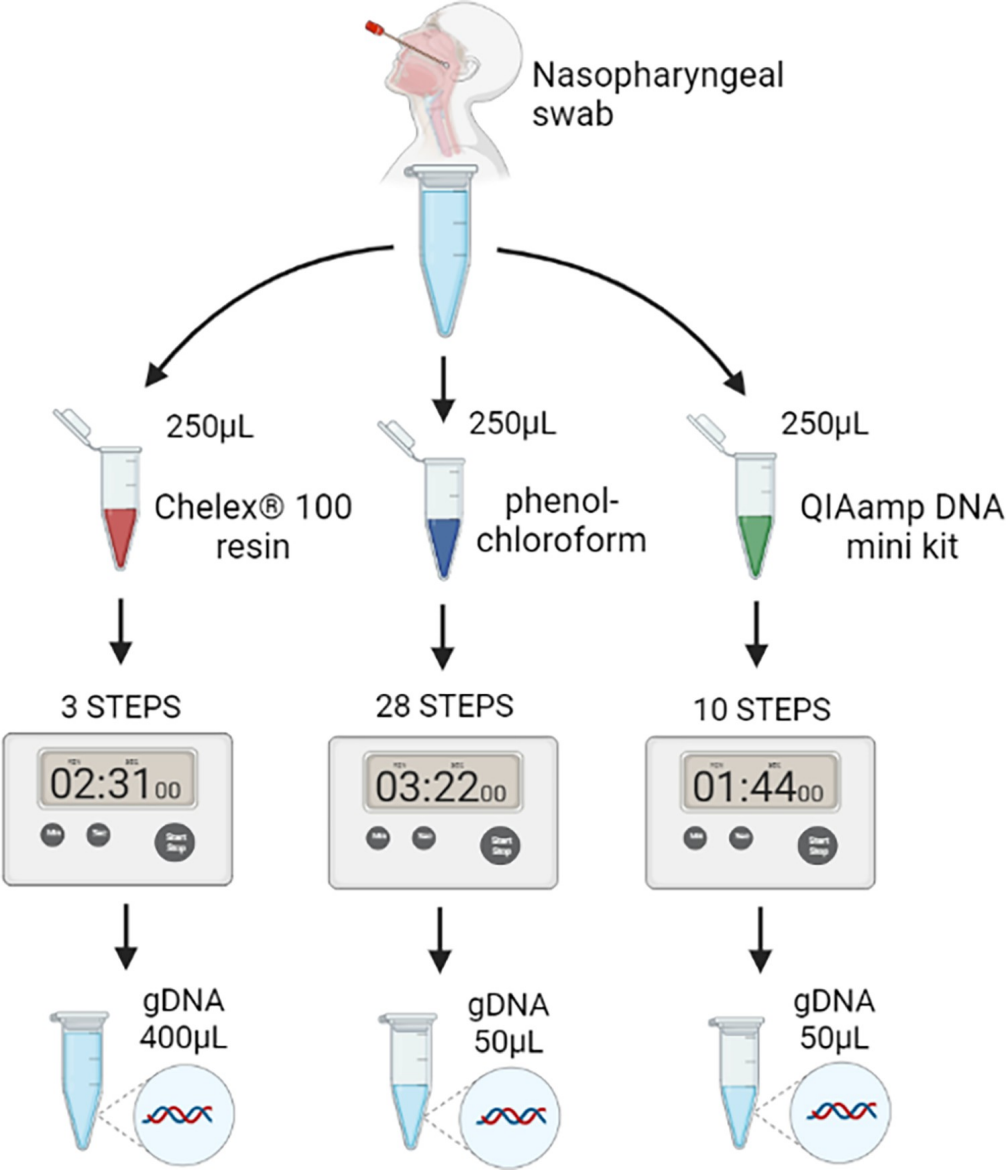
Nasopharyngeal swab collection is the gold standard for diagnosis of SARS-CoV-2 and this sample can be used for DNA extraction for genetic analyses. DNA extraction can be performed by different methods such as Chelex^®^100, phenol-chloroform, and commercial kits. Such methods differ in terms of principle, number of steps, time to perform the technique and yield of the extracted material.

Initially, each protocol was compared with respect to some technical-methodological criteria (Fig 1).

In relation to steps number, the Chelex^®^100 protocol presented the shortest steps number (3 steps), followed by QIAamp DNA Mini Kit (10 steps) and phenol-chloroform (28 steps). Regarding protocol runtime, the QIAamp DNA mini kit showed the shortest time for extraction of 24 samples (1 hour and 44 minutes), followed by Chelex®100 (2 hours and 31 minutes) and phenol-chloroform (3 hours and 22 minutes). We chose this sample number because it allows comfortable manual DNA extraction using conventional tabletop centrifuges found in most labs. When compared to the total retrieved volume, the Chelex^®^100 protocol was able to recover the largest DNA volume (400μL) compared to QIAamp DNA mini kit (50μL) and phenol-chloroform (50μL).

We then compared the costs for consumables used in the different methods (Table 1).

**Table 1:** Costs associated with the DNA genomic extraction by several methods, per one reaction (costs are based on Brazilian reagent prices at time of the study and are converted to US$).

Consumables	Costs per Sample Extracted		
	Chelex^®^100 resin	Phenol-chloroform	QIAamp DNA Mini Kit
Chelex^®^100 sodium form	0.001	-	-
QIAamp DNA mini kit (250 reations)	-	-	4.779
Phenol solution	-	0.171	
Pipet tips (200 μL)	0.039	0.097	0.039
Pipet Tips (1000 μL)	0.043	0.214	0.171
Proteinase K	-	0.970	-
Microcentrifuge tube (0.5 mL)	0.031	-	-
Microcentrifuge tube (2 mL)	0.039	0.155	0.077
Several chemical reagents*	-	0.343	
**Price (US $)**	**0.153**	**1.950**	**5.066**

*Reagents used in the phenol-chloroform extraction: magnesium chloride, sodium chloride, sodium acetate, chloroform, ethanol PA, amyl alcohol, tris(hydroxymethyl)aminomethane hydrochloride, sodium dodecyl sulfate, ethylenedinitrilotetraacetic acid.

The Chelex^®^100 method gave the lowest cost (US$ 0.153), followed by phenol-chloroform (US$ 1.950) and the QIAamp DNA mini kit (US$ 5.066). Thus, the Chelex^®^100 was found to be 33 times cheaper than a commercial kit and almost 13 times cheaper than a classical phenol-chloroform method.

A greater number of steps implies greater human interference, including tube change/disposal, resulting in increased chances of DNA contamination- as well as increased amount of waste, higher costs, exposure to harmful chemicals (phenol, chloroform, isoamyl alcohol) and ergonomic risk [11, 17].

**Fig 2:**
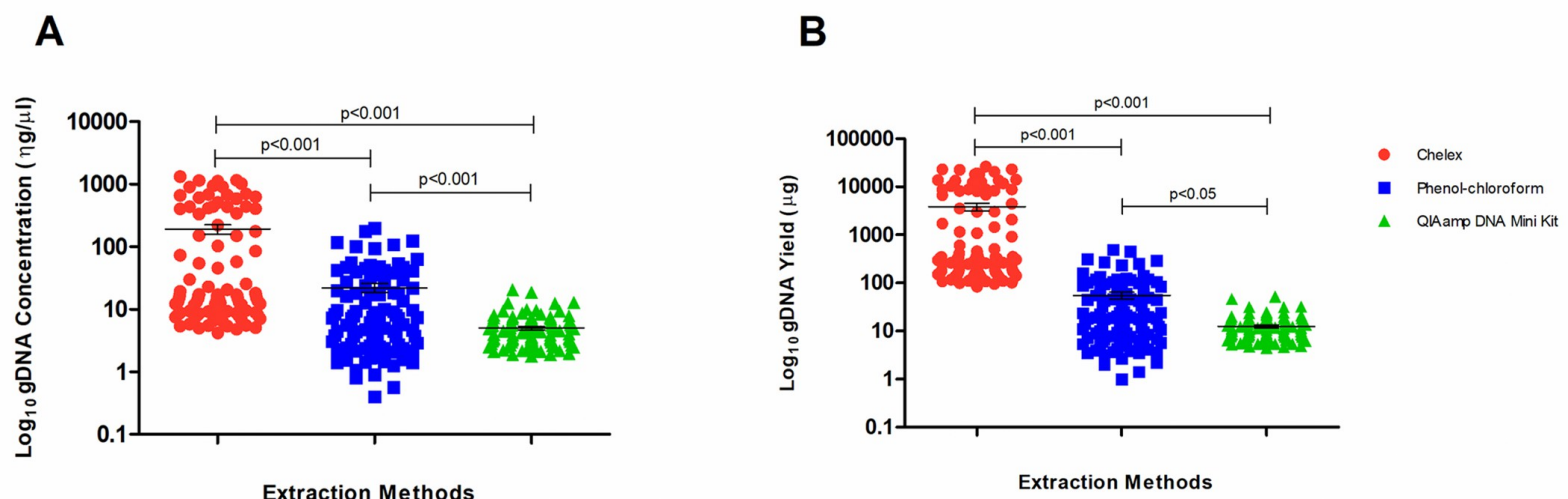
Genomic DNA concentration and yield according to extraction methods. A) DNA concentration; B) DNA yield. DNA extraction methods were compared by Kruskal-Wallis Test with Dunn’s Multiple Comparison.

The concentration and yield of the DNA obtained by the three extraction methods using nasopharyngeal swab samples of COVID-19 individuals are shown in Fig 2.

The DNA concentration was significantly higher (media = 191.8 ng/μl, standard error of the mean–SEM = 32.6 ng/μl) for the Chelex^®^100 protocol than for the phenol-chloroform method (mean = 22.1 ng/μl, SEM = 3.5 ng/μl, p<0.0001) and QIAamp DNA mini kit (mean = 5.02 ng/μl, SEM = 0.3 ng/μl, p<0.0001). In addition, the phenol-chloroform present a higher DNA concentration (p<0.0001) than the QIAamp DNA mini kit (Fig 2A). In relation to total DNA yield, samples extracted by Chelex^®^100 protocol produced a higher yield (mean = 3836μg, SEM = 652.8μg) than samples processed using phenol-chloroform (mean = 55.22μg, SEM = 8.8μg, p<0.0001) or QIAamp DNA mini kit (mean = 12.55μg, SEM = 0.8μg, p<0.0001) methods. The phenol-chloroform gave a higher DNA yield (p<0.05) than the QIAamp DNA mini kit (Fig 2B).

**Fig 3:**
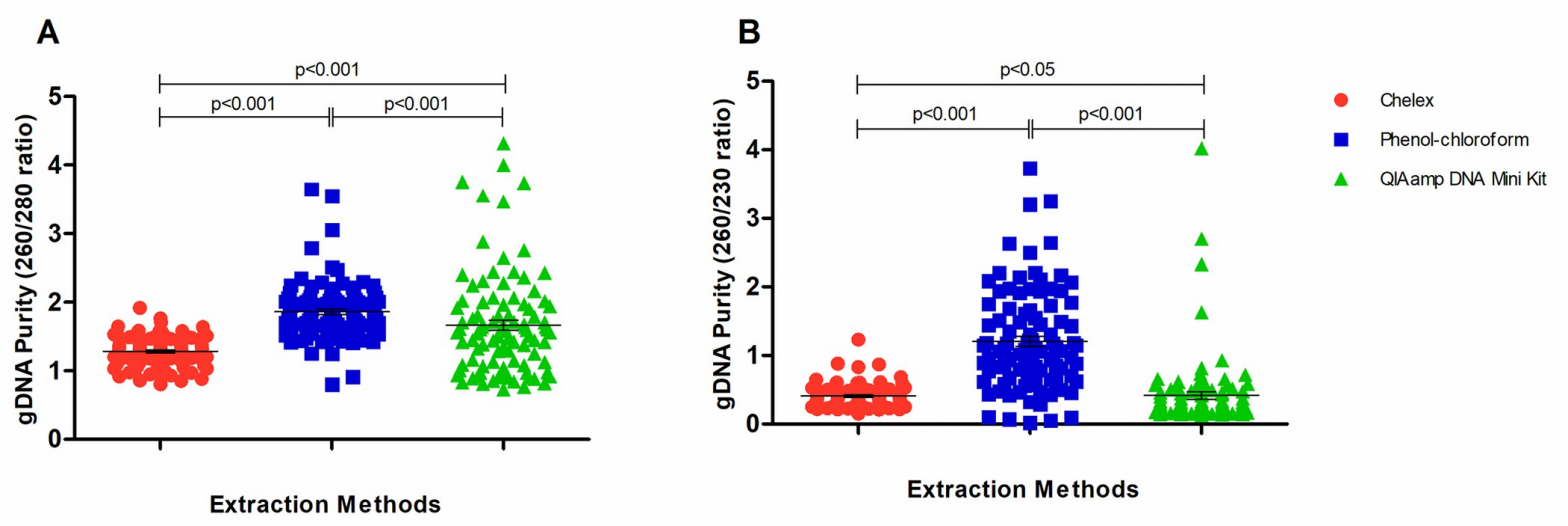
Genomic DNA purity measured by spectrophotometer. A) 260/280 ratio; B) 260/230 ratio. The DNA ratio obtained by different extraction methods were compared by Kruskal-Wallis Test with Dunn’s Multiple Comparison.

The DNA purities, as determined spectrophotometrically by the 260/280 and 260/230 ratios, for three extraction methods are shown in Fig 3.

The Chelex^®^100 protocol showed the lowest 260/280 ratio (mean = 1.284, SEM = 0.02), differing significantly from phenol-chloroform (mean = 1.861; SEM = 0.04; p<0.0001) and QIAamp DNA mini kit (mean = 1.663; SEM = 0.07; p<0.0001) purifications. A statistically significantly difference was found between the phenol-chloroform and QIAamp DNA mini kit (p<0.0001) (Fig 3A) with respect to this parameter. For the 260/230 ratio, the phenol-chloroform method gave the highest value (mean = 1.207; SEM = 0.07), significantly differing from the DNA extracted by Chelex^®^100 (mean = 0.4107; SEM = 0.02; p<0.0001) and QIAamp DNA mini kit (mean = 0.4167; SEM = 0.05; p<0.0001). Significantly differing also was observed between QIAamp DNA mini kit and Chelex^®^100 (p<0.05) (Fig 3B). The 260/230 ratio indicates the purity of the nucleic acid sample from salts and other contaminants which can absorb at 230 nm. Since proteins absorb light strongly at 280 nm wavelength, a low 260/280 ratio indicates the presence of high amounts of protein, relative to nucleic acids. The phenol-chloroform had the best DNA quality in comparison to Chelex^®^100 and QIAamp DNA mini kit as measured by the 260/280 and 260/230 ratios. However, the phenol-chloroform uses highly toxic reagents and is labour intense and time consuming [29].

The literature shows a great deal of variability in DNA concentrations, yield and quality of the Chelex^®^100 method depending on the sample type used [18, 19, 21, 29–31]. Some studies using blood samples [21], saliva placed onto cotton swabs and air-dried [21], semen [21, 29], human hair [19] and cigarette butts [22]. We have reported higher quantity and yield of DNA extracted with Chelex^®^100, but lower quality in some cases as we observed in this study.

**Fig 4:**
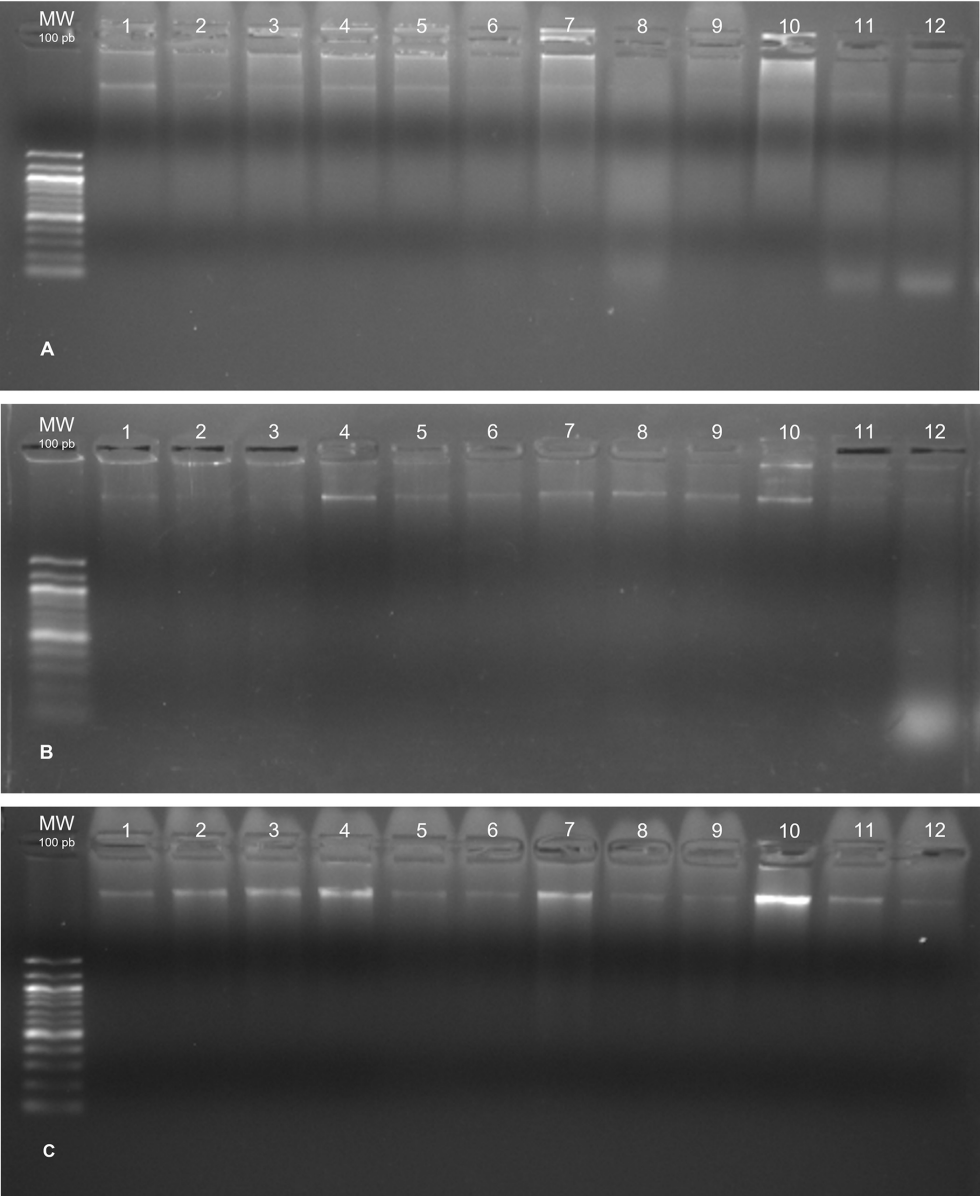
Genomic DNA quality according extraction methods measured by 1.5% agarose gel. A) Chelex^®^100; B) Phenol-Chloroform; C) QIAamp DNA mini kit. Legend: MW = molecular weight.

In our nasopharyngeal samples, the DNA integrity was also evaluated by running samples in 1.5% agarose gel and the results are shown in Fig 4.

**Fig 5:**
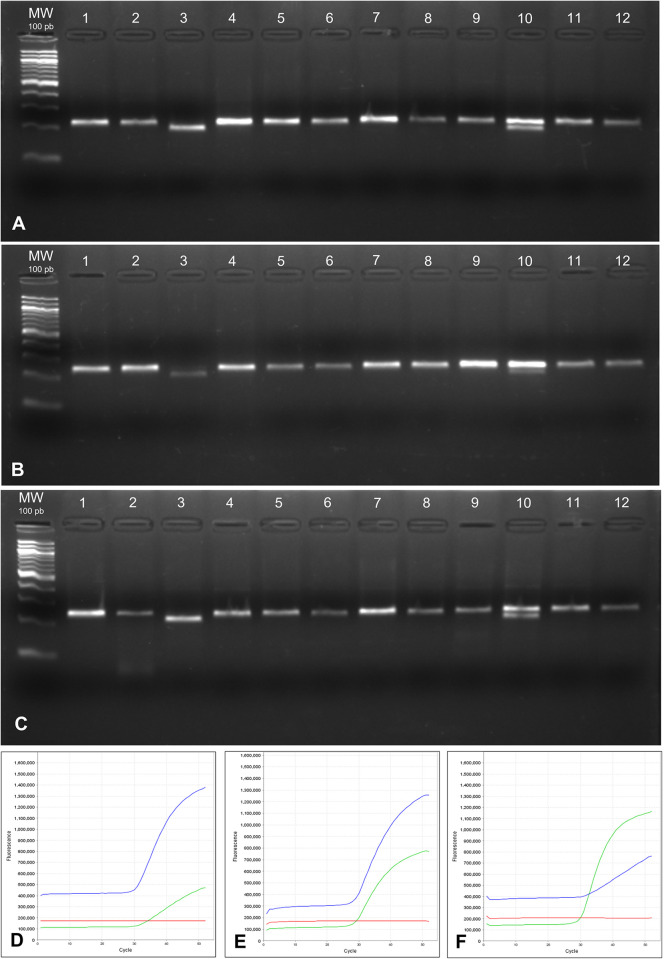
Applications of genomic DNA extracted by different methods in PCR RFLP and Real Time PCR with allelic specific probes (TaqMan) for SNP rs181133 in *MTHFR* gene. A, B, C– 3% Agarose gel of amplicons referent to PCR RFLP; D, E, F–Real time PCR curves. A–Genomic DNA extracted by chelex; B–Genomic DNA extracted by phenol-chloroform; C–Genomic DNA extracted by QIAamp DNA blood mini kit. Lane 1, 2, 4, 5, 6, 7, 8, 9, 11 and 12 –Genotype G/G; Lane 3 –Genotype A/A; Lane 10 –Genotype G/A; D–Curve of real time PCR referent to genotype A/A (sample 3); E–Curve of real time PCR referent to genotype G/A (sample 10); F–Curve of real time PCR referent to genotype G/G (Sample 1, 2, 4, 5, 6, 7, 8, 9, 11 and 12). The lines green, blue and red represents, respectively, the fluorescent dye VIC (allele A), FAM (allele G) and ROX (passive reference dye).

The agarose gel analysis revealed uniformity of the samples extracted by the Chelex^®^100 method (Fig 4A), phenol-chloroform (Fig 4B) and the QIAamp DNA mini kit (Fig 4C) in relation to the integrity of the genomic DNA, with no evident degradation. The differences of DNA quality seen in the different methods did not seem to interfere with the success of amplification using two different genotyping methodologies, RFLP and qRT-PCR (Fig 5).

In the RFLP assays, samples extracted by three different protocols showed uniform amplification and were properly cut by the restriction enzyme used to detect the SNP (Fig 5A–5C). For qRT-PCR using allele-specific probes, the success of amplification was considered normal, allowing the correct genotyping of the tested SNP (Fig 5D–5F). In both genotyping methodologies, no divergence was found regardless of the DNA extraction method used, a fact that corroborates that DNA obtained with these methods can be successfully genotyped.

The success of DNA amplification by both PCR methodologies in our study differed from reports suggesting that samples extracted by Chelex^®^100 or phenol-chloroform may have PCR inhibitors [18]. PCR inhibition in Chelex^®^100-extracted DNA seems to be associated with the type of biological sample used [31]. In blood samples, the removal of heme groups by Chelex^®^100 resin was reported to be inefficient, resulting in PCR inhibition [31]. In addition, some studies have suggested that the DNA extracted by Chelex^®^100 would degrade the DNA, which would make it unsuitable for RFLP analysis [18], which was also not supported by our results.

## Conclusions

Taken together, our results suggest that in nasopharyngeal samples of COVID-19 patients, the use of QIAamp DNA mini kit is limited by its low DNA quantity and quality, as well high cost per sample. On the other hand, the phenol-chloroform-based extraction is a laborious and potentially hazardous, despite the higher level of DNA purity observed. In contrast, Chelex^®^100 resin emerged as an affordable, effective, fast, and simple method, which can be carried out in few steps. The method does not require the use of organic solvents and manipulation steps, showing a low risk of sample contamination and offers lower hazard risk to the operator and the environment. Thus, the Chelex^®^100 method is a cheap, safe, simple, fast, and effective method for the DNA extraction from nasopharyngeal samples of COVID-19 patients suitable for developing countries and minimal settings.

## Supporting information

S1 Table Genomic DNA extracted of nasopharyngeal swab samples of COVID-19 individual by different extraction methods. (DOCX)

S2 Table Runtime for extracting 24 nasopharyngeal swab samples of COVID-19 individuals by different methods. (DOCX)

S3 Table *MTHFR* genotyping (rs181133) using different PCR methodologies. (DOCX)

S1 Raw images (PDF)
