# Correction: Comparison of DNA extraction methods for COVID-19 host genetics studies

**DOI:** 10.1371/journal.pone.0314215

**Published:** 2024-11-15

**Authors:** Ronaldo Celerino da Silva, Suelen Cristina de Lima, Wendell Palôma Maria dos Santos Reis, Jurandy Júnior Ferraz de Magalhães, Ronaldo Nascimento de Oliveira, Brijesh Rathi, Alain Kohl, Marcos André Cavalcanti Bezerra, Lindomar Pena

The fifth author’s name is written incorrectly. The correct name is: Ronaldo Nascimento de Oliveira. The correct citation is: Silva RCd, de Lima SC, dos Santos Reis WPM, de Magalhães JJF, de Oliveira RN, Rathi B, et al. (2023) Comparison of DNA extraction methods for COVID-19 host genetics studies. PLoS ONE 18(10): e0287551. https://doi.org/10.1371/journal.pone.0287551.
