# Supplementary material for: Comparison of DNA extraction methods for COVID-19 host genetics studies
Source: PLoS One. 2023 Oct 30;18(10):e0287551. doi: 10.1371/journal.pone.0287551 (PMC10615309; doi:10.1371/journal.pone.0287551)
Supplement: S1 Table — (DOCX) [file pone.0287551.s001.docx]

**S1 Table.** Genomic DNA extracted of Nasopharyngeal swab samples of COVID-19 individual by different extraction methods.

| **Research** | **Sample ID** | **Chelex®100** | | | | **QIAamp DNA Mini Kit** | | | | **Phenol-Chloroform** | | | |
| --- | --- | --- | --- | --- | --- | --- | --- | --- | --- | --- | --- | --- | --- |
|  |  | **Concentration (ng/µl)** | **Yield (ug)** | **260/280 ratio** | **260/230 ratio** | **Concentration (ng/µl)** | **Yield (ug)** | **260/280 ratio** | **260/230 ratio** | **Concentration (ng/µl)** | **Yield (ug)** | **260/280**  **ratio** | **260/230**  **ratio** |
| 1 | 1 | 627.833 | 12556.667 | 1.320 | 0.883 | 5.800 | 14.500 | 3.757 | 0.310 | 13.700 | 34.250 | 1.963 | 0.673 |
| 1 | 2 | 442.100 | 8842.000 | 1.180 | 0.650 | 4.900 | 12.250 | 2.647 | 0.427 | 9.433 | 23.583 | 2.030 | 0.837 |
| 1 | 3 | 435.900 | 8718.000 | 1.220 | 0.593 | 5.533 | 13.833 | 2.000 | 0.587 | 18.300 | 45.750 | 1.983 | 1.233 |
| 1 | 4 | 667.067 | 13341.333 | 1.317 | 0.537 | 4.600 | 11.500 | 2.887 | 0.447 | 7.467 | 18.667 | 2.247 | 0.917 |
| 1 | 5 | 680.400 | 13608.000 | 1.370 | 0.330 | 4.867 | 12.167 | 1.920 | 0.260 | 12.900 | 32.250 | 1.707 | 0.647 |
| 1 | 6 | 687.167 | 13743.333 | 1.340 | 0.330 | 2.800 | 7.000 | 3.470 | 0.320 | 4.767 | 11.917 | 2.217 | 1.977 |
| 1 | 7 | 701.800 | 14036.000 | 1.367 | 0.340 | 7.900 | 19.750 | 2.023 | 0.597 | 18.267 | 45.667 | 2.000 | 0.837 |
| 1 | 8 | 1027.733 | 20554.667 | 1.460 | 0.450 | 6.200 | 15.500 | 2.247 | 0.560 | 37.833 | 94.583 | 2.063 | 2.070 |
| 1 | 9 | 177.167 | 3543.333 | 1.230 | 0.350 | 4.933 | 12.333 | 2.067 | 0.287 | 20.000 | 50.000 | 1.843 | 1.307 |
| 1 | 10 | 151.333 | 3026.667 | 1.187 | 0.310 | 8.367 | 20.917 | 2.063 | 0.927 | 17.433 | 43.583 | 1.807 | 3.203 |
| 1 | 11 | 942.900 | 18858.000 | 1.567 | 0.520 | 5.433 | 13.583 | 2.363 | 0.447 | 45.967 | 114.917 | 2.140 | 1.947 |
| 1 | 12 | 402.400 | 8048.000 | 1.203 | 0.537 | 5.400 | 13.500 | 2.443 | 0.507 | 56.100 | 140.250 | 2.143 | 1.660 |
| 1 | 13 | 20.633 | 412.667 | 1.167 | 0.317 | 6.233 | 15.583 | 2.400 | 0.297 | 3.733 | 9.333 | 2.027 | 2.207 |
| 1 | 14 | 17.400 | 348.000 | 1.197 | 0.293 | 5.733 | 14.333 | 2.760 | 0.253 | 2.900 | 7.250 | 1.940 | 1.683 |
| 1 | 15 | 1131.733 | 22634.667 | 1.553 | 0.530 | 12.933 | 32.333 | 2.013 | 0.730 | 42.033 | 105.083 | 2.090 | 2.110 |
| 1 | 16 | 4.200 | 84.000 | 0.880 | 0.230 | 3.267 | 8.167 | 1.963 | 0.163 | 2.500 | 6.250 | 1.243 | 0.103 |
| 1 | 17 | 5.833 | 116.667 | 1.253 | 0.380 | 3.100 | 7.750 | 2.283 | 0.163 | 0.567 | 1.417 | 0.803 | 0.053 |
| 1 | 18 | 5.533 | 110.667 | 1.247 | 0.300 | 3.400 | 8.500 | 3.740 | 0.187 | 1.700 | 4.250 | 0.910 | 0.020 |
| 1 | 19 | 8.533 | 170.667 | 1.463 | 0.470 | 5.567 | 13.917 | 3.557 | 0.260 | 1.400 | 3.500 | 1.700 | 0.067 |
| 1 | 20 | 16.633 | 332.667 | 1.277 | 0.413 | 4.700 | 11.750 | 4.317 | 0.240 | 2.167 | 5.417 | 2.233 | 1.913 |
| 1 | 21 | 13.667 | 273.333 | 1.583 | 0.387 | 9.567 | 23.917 | 1.967 | 0.460 | 3.800 | 9.500 | 2.243 | 2.140 |
| 1 | 22 | 12.267 | 245.333 | 1.917 | 0.687 | 9.867 | 24.667 | 1.707 | 0.403 | 18.100 | 45.250 | 1.823 | 1.773 |
| 1 | 23 | 17.200 | 344.000 | 1.453 | 0.370 | 6.767 | 16.917 | 1.640 | 0.417 | 7.167 | 17.917 | 2.013 | 2.087 |
| 1 | 24 | 8.400 | 168.000 | 1.450 | 0.320 | 3.967 | 9.917 | 1.420 | 0.260 | 3.400 | 8.500 | 1.660 | 1.607 |
| 1 | 25 | 16.633 | 332.667 | 1.507 | 0.527 | 2.167 | 5.417 | 0.843 | 0.170 | 2.733 | 6.833 | 2.077 | 0.587 |
| 1 | 26 | 4.967 | 99.333 | 1.233 | 0.320 | 1.900 | 4.750 | 1.160 | 0.147 | 3.067 | 7.667 | 1.803 | 0.430 |
| 1 | 27 | 12.767 | 255.333 | 1.647 | 0.367 | 2.533 | 6.333 | 1.787 | 0.177 | 1.267 | 3.167 | 3.057 | 0.577 |
| 1 | 28 | 22.933 | 458.667 | 1.490 | 0.400 | 4.500 | 11.250 | 1.560 | 0.307 | 5.700 | 14.250 | 2.080 | 1.487 |
| 1 | 29 | 16.600 | 332.000 | 1.553 | 0.413 | 2.167 | 5.417 | 0.973 | 0.157 | 2.300 | 5.750 | 2.793 | 1.453 |
| 1 | 30 | 8.533 | 170.667 | 1.287 | 0.287 | 3.400 | 8.500 | 1.083 | 0.243 | 1.600 | 4.000 | 2.293 | 1.167 |
| 1 | 31 | 1148.633 | 22972.667 | 1.533 | 0.513 | 6.500 | 16.250 | 1.320 | 0.657 | 117.200 | 293.000 | 2.077 | 1.937 |
| 1 | 32 | 8.733 | 174.667 | 1.763 | 0.373 | 2.200 | 5.500 | 4.003 | 0.153 | 2.000 | 5.000 | 1.797 | 0.813 |
| 1 | 33 | 11.933 | 238.667 | 1.253 | 0.243 | 2.467 | 6.167 | 1.810 | 0.167 | 2.400 | 6.000 | 1.700 | 0.417 |
| 1 | 34 | 15.300 | 306.000 | 1.483 | 0.413 | 5.400 | 13.500 | 2.163 | 0.320 | 7.200 | 18.000 | 1.987 | 1.147 |
| 2 | 35 | 9.200 | 184.000 | 1.280 | 0.493 | 3.667 | 9.167 | 1.010 | 0.207 | 4.433 | 11.083 | 1.540 | 0.880 |
| 2 | 36 | 612.533 | 12250.667 | 1.320 | 0.870 | 3.233 | 8.083 | 0.907 | 0.333 | 42.233 | 105.583 | 1.683 | 0.960 |
| 2 | 37 | 150.167 | 3003.333 | 1.330 | 0.373 | 5.700 | 14.250 | 1.070 | 0.530 | 93.533 | 233.833 | 2.043 | 1.753 |
| 2 | 38 | 57.433 | 1148.667 | 1.360 | 0.460 | 18.600 | 46.500 | 1.597 | 0.813 | 197.067 | 492.667 | 2.030 | 2.643 |
| 2 | 39 | 7.767 | 155.333 | 1.363 | 0.440 | 3.433 | 8.583 | 0.923 | 0.200 | 2.367 | 5.917 | 1.740 | 1.180 |
| 2 | 40 | 515.533 | 10310.667 | 1.220 | 0.340 | 3.367 | 8.417 | 0.887 | 0.513 | 38.367 | 95.917 | 1.703 | 1.493 |
| 2 | 41 | 333.233 | 6664.667 | 1.227 | 0.403 | 3.467 | 8.667 | 0.893 | 0.330 | 33.533 | 83.833 | 1.830 | 1.060 |
| 2 | 42 | 438.600 | 8772.000 | 1.230 | 0.607 | 3.833 | 9.583 | 0.970 | 0.373 | 124.533 | 311.333 | 1.763 | 1.177 |
| 2 | 43 | 7.833 | 156.667 | 1.250 | 0.413 | 3.100 | 7.750 | 1.760 | 0.210 | 1.567 | 3.917 | 1.733 | 0.503 |
| 2 | 44 | 18.233 | 364.667 | 1.537 | 0.507 | 2.967 | 7.417 | 0.897 | 0.180 | 1.400 | 3.500 | 1.540 | 1.733 |
| 2 | 45 | 8.133 | 162.667 | 1.240 | 0.250 | 2.667 | 6.667 | 0.810 | 0.157 | 108.833 | 272.083 | 1.803 | 0.787 |
| 2 | 46 | 408.767 | 8175.333 | 1.187 | 0.597 | 2.700 | 6.750 | 0.833 | 0.260 | 18.600 | 46.500 | 2.003 | 1.060 |
| 2 | 47 | 223.200 | 4464.000 | 1.220 | 0.400 | 2.467 | 6.167 | 0.763 | 0.280 | 22.000 | 55.000 | 1.807 | 0.893 |
| 2 | 48 | 7.833 | 156.667 | 1.330 | 0.383 | 3.100 | 7.750 | 0.907 | 0.180 | 54.000 | 135.000 | 1.683 | 0.953 |
| 2 | 49 | 103.667 | 2073.333 | 0.993 | 0.290 | 5.067 | 12.667 | 1.127 | 0.300 | 27.600 | 69.000 | 2.510 | 0.847 |
| 2 | 50 | 13.100 | 262.000 | 1.497 | 0.500 | 4.900 | 12.250 | 1.087 | 0.277 | 3.667 | 9.167 | 1.663 | 1.310 |
| 2 | 51 | 13.167 | 263.333 | 0.860 | 0.153 | 5.700 | 14.250 | 1.150 | 0.257 | 17.367 | 43.417 | 1.757 | 1.550 |
| 2 | 52 | 7.033 | 140.667 | 1.403 | 0.357 | 3.500 | 8.750 | 1.010 | 0.133 | 1.467 | 3.667 | 1.767 | 1.063 |
| 2 | 53 | 5.100 | 102.000 | 1.097 | 0.247 | 2.500 | 6.250 | 0.733 | 0.143 | 0.400 | 1.000 | 1.253 | 3.733 |
| 2 | 54 | 906.867 | 18137.333 | 1.537 | 0.507 | 7.533 | 18.833 | 1.423 | 0.540 | 179.400 | 448.500 | 1.957 | 2.633 |
| 2 | 55 | 15.367 | 307.333 | 1.033 | 0.260 | 3.267 | 8.167 | 0.930 | 0.193 | 8.133 | 20.333 | 1.693 | 1.110 |
| 2 | 56 | 19.267 | 385.333 | 1.033 | 0.230 | 8.633 | 21.583 | 1.287 | 0.477 | 4.700 | 11.750 | 1.540 | 0.863 |
| 2 | 57 | 25.467 | 509.333 | 1.077 | 0.267 | 9.267 | 23.167 | 1.350 | 0.497 | 48.200 | 120.500 | 1.753 | 1.187 |
| 2 | 58 | 9.600 | 192.000 | 1.503 | 0.403 | 3.367 | 8.417 | 0.920 | 0.193 | 40.167 | 100.417 | 1.623 | 0.773 |
| 2 | 59 | 9.333 | 186.667 | 1.470 | 0.290 | 7.367 | 18.417 | 1.253 | 0.337 | 7.033 | 17.583 | 1.813 | 2.503 |
| 2 | 60 | 12.300 | 246.000 | 1.443 | 0.347 | 3.867 | 9.667 | 1.043 | 0.240 | 10.067 | 25.167 | 1.637 | 1.433 |
| 2 | 61 | 73.033 | 1460.667 | 1.450 | 0.603 | 2.633 | 6.583 | 0.820 | 0.170 | 1.067 | 2.667 | 1.823 | 0.467 |
| 2 | 62 | 8.233 | 164.667 | 1.223 | 0.383 | 3.433 | 8.583 | 0.940 | 0.193 | 0.800 | 2.000 | 1.700 | 0.437 |
| 2 | 63 | 6.500 | 130.000 | 1.710 | 0.460 | 3.200 | 8.000 | 0.930 | 0.193 | 7.333 | 18.333 | 1.443 | 0.560 |
| 2 | 64 | 7.600 | 152.000 | 1.267 | 0.443 | 2.967 | 7.417 | 0.977 | 0.167 | 0.900 | 2.250 | 3.647 | 0.323 |
| 2 | 65 | 16.900 | 338.000 | 1.493 | 0.440 | 9.367 | 23.417 | 1.423 | 0.570 | 41.400 | 103.500 | 1.870 | 2.167 |
| 2 | 66 | 8.900 | 178.000 | 1.260 | 0.360 | 12.433 | 31.083 | 1.447 | 0.620 | 29.400 | 73.500 | 1.813 | 1.510 |
| 2 | 67 | 17.167 | 343.333 | 1.227 | 0.403 | 2.900 | 7.250 | 1.610 | 0.213 | 7.767 | 19.417 | 1.623 | 1.217 |
| 3 | 68 | 6.900 | 138.000 | 1.063 | 0.223 | 2.600 | 6.500 | 1.130 | 0.193 | 2.367 | 5.917 | 1.813 | 0.477 |
| 3 | 69 | 6.767 | 135.333 | 1.177 | 0.217 | 1.867 | 4.667 | 0.853 | 0.143 | 1.667 | 4.167 | 2.277 | 0.963 |
| 3 | 70 | 344.033 | 6880.667 | 1.190 | 0.453 | 6.800 | 17.000 | 1.607 | 0.217 | 16.167 | 40.417 | 1.537 | 0.607 |
| 3 | 71 | 16.267 | 325.333 | 1.157 | 0.280 | 3.467 | 8.667 | 1.377 | 0.430 | 5.033 | 12.583 | 2.007 | 0.623 |
| 3 | 72 | 589.433 | 11788.667 | 1.323 | 0.833 | 5.133 | 12.833 | 1.633 | 0.367 | 47.667 | 119.167 | 1.477 | 0.967 |
| 3 | 73 | 15.333 | 306.667 | 1.200 | 0.367 | 5.533 | 13.833 | 1.587 | 0.230 | 9.800 | 24.500 | 1.410 | 1.440 |
| 3 | 74 | 12.267 | 245.333 | 1.577 | 0.440 | 2.933 | 7.333 | 1.640 | 0.483 | 3.133 | 7.833 | 1.433 | 1.940 |
| 3 | 75 | 911.567 | 18231.333 | 1.560 | 0.520 | 7.033 | 17.583 | 1.707 | 0.330 | 63.633 | 159.083 | 1.980 | 1.973 |
| 3 | 76 | 9.833 | 196.667 | 1.037 | 0.213 | 7.333 | 18.333 | 1.677 | 0.153 | 7.633 | 19.083 | 1.420 | 0.843 |
| 3 | 77 | 415.633 | 8312.667 | 1.190 | 1.233 | 12.633 | 31.583 | 1.603 | 0.587 | 24.267 | 60.667 | 2.010 | 0.797 |
| 3 | 78 | 30.133 | 602.667 | 1.180 | 0.250 | 4.600 | 11.500 | 1.550 | 0.273 | 7.867 | 19.667 | 1.903 | 0.933 |
| 3 | 79 | 434.833 | 8696.667 | 1.227 | 0.610 | 5.533 | 13.833 | 1.480 | 0.533 | 50.633 | 126.583 | 1.757 | 0.637 |
| 3 | 80 | 1329.833 | 26596.667 | 1.587 | 0.550 | 4.967 | 12.417 | 1.667 | 1.627 | 101.600 | 254.000 | 1.963 | 1.353 |
| 3 | 81 | 85.700 | 1714.000 | 0.920 | 0.270 | 7.600 | 19.000 | 1.503 | 0.243 | 8.367 | 20.917 | 1.477 | 0.930 |
| 3 | 82 | 54.600 | 1092.000 | 1.050 | 0.260 | 20.667 | 51.667 | 1.713 | 0.657 | 47.000 | 117.500 | 1.520 | 3.253 |
| 3 | 83 | 11.733 | 234.667 | 1.120 | 0.413 | 2.233 | 5.583 | 1.943 | 0.157 | 6.200 | 15.500 | 1.457 | 0.937 |
| 3 | 84 | 14.433 | 288.667 | 1.127 | 0.300 | 4.533 | 11.333 | 2.310 | 0.287 | 5.667 | 14.167 | 1.557 | 1.173 |
| 3 | 85 | 1155.500 | 23110.000 | 1.540 | 0.530 | 3.033 | 7.583 | 1.713 | 0.190 | 25.967 | 64.917 | 2.200 | 2.200 |
| 3 | 86 | 6.467 | 129.333 | 0.950 | 0.407 | 3.467 | 8.667 | 1.387 | 2.703 | 9.767 | 24.417 | 1.427 | 1.127 |
| 3 | 87 | 14.800 | 296.000 | 0.930 | 0.213 | 4.400 | 11.000 | 1.440 | 0.283 | 9.000 | 22.500 | 1.573 | 1.020 |
| 3 | 88 | 5.367 | 107.333 | 1.510 | 0.243 | 2.100 | 5.250 | 1.560 | 2.333 | 9.233 | 23.083 | 2.350 | 0.093 |
| 3 | 89 | 13.700 | 274.000 | 0.983 | 0.227 | 3.267 | 8.167 | 1.610 | 0.460 | 4.533 | 11.333 | 2.067 | 0.287 |
| 3 | 90 | 5.367 | 107.333 | 0.987 | 0.397 | 1.967 | 4.917 | 1.293 | 0.717 | 2.367 | 5.917 | 1.830 | 1.177 |
| 3 | 91 | 17.033 | 340.667 | 1.383 | 0.470 | 3.367 | 8.417 | 1.800 | 0.177 | 4.000 | 10.000 | 1.537 | 1.087 |
| 3 | 92 | 45.867 | 917.333 | 0.970 | 0.210 | 2.467 | 6.167 | 1.510 | 4.023 | 4.300 | 10.750 | 2.297 | 0.473 |
| 3 | 93 | 16.400 | 328.000 | 1.387 | 0.473 | 8.067 | 20.167 | 1.593 | 0.280 | 3.667 | 9.167 | 3.550 | 1.980 |
| 3 | 94 | 7.500 | 150.000 | 1.143 | 0.480 | 4.200 | 10.500 | 1.660 | 0.253 | 2.633 | 6.583 | 1.973 | 0.623 |
| 3 | 95 | 13.133 | 262.667 | 1.007 | 0.250 | 4.000 | 10.000 | 1.643 | 0.197 | 35.333 | 88.333 | 1.440 | 1.097 |
| 3 | 96 | 4.800 | 96.000 | 0.807 | 0.337 | 2.133 | 5.333 | 1.903 | 0.157 | 2.167 | 5.417 | 1.470 | 1.143 |
| 3 | 97 | 9.400 | 188.000 | 1.033 | 0.307 | 2.267 | 5.667 | 2.433 | 0.140 | 2.100 | 5.250 | 1.547 | 0.707 |
| 3 | 98 | 5.500 | 110.000 | 0.850 | 0.340 | 1.767 | 4.417 | 2.440 | 0.137 | 2.767 | 6.917 | 1.423 | 0.963 |
| 3 | 99 | 9.633 | 192.667 | 1.640 | 0.540 | 7.033 | 17.583 | 1.807 | 0.173 | 1.433 | 3.583 | 2.473 | 0.457 |
| 3 | 100 | 8.767 | 175.333 | 0.977 | 0.350 | 3.533 | 8.833 | 1.447 | 0.250 | 4.533 | 11.333 | 1.707 | 0.617 |
