# Supplementary material for: Comparison of DNA extraction methods for COVID-19 host genetics studies
Source: PLoS One. 2023 Oct 30;18(10):e0287551. doi: 10.1371/journal.pone.0287551 (PMC10615309; doi:10.1371/journal.pone.0287551)
Supplement: S2 Table — (DOCX) [file pone.0287551.s002.docx]

**S2 Table.** Runtime for extracting 24 nasopharyngeal swab samples of COVID-19 individuals by different methods.

| **Research** | **Protocol routime** | | |
| --- | --- | --- | --- |
|  | **Chelex®100** | **QIAamp DNA Mini Kit** | **Phenol-Chloroform** |
| 1 | 2h 24 min | 1h 42min | 3h 32 min |
| 2 | 2h 34 min | 1h 53 min | 3h 10 min |
| 3 | 2h 35 min | 1h 37min | 3h 25 min |
| **Median** | **2h 31 min** | **1h 44 min** | **3h 22min** |
