# Supplementary material for: Comparison of DNA extraction methods for COVID-19 host genetics studies
Source: PLoS One. 2023 Oct 30;18(10):e0287551. doi: 10.1371/journal.pone.0287551 (PMC10615309; doi:10.1371/journal.pone.0287551)
Supplement: S3 Table — (DOCX) [file pone.0287551.s003.docx]

**S3 Table.** *MTHFR* genotyping (rs181133) using different PCR methodologies.

| **Extration Methods** | **Aplications** | **Samples** | | | | | | | | | | | | | | | | | | | |
| --- | --- | --- | --- | --- | --- | --- | --- | --- | --- | --- | --- | --- | --- | --- | --- | --- | --- | --- | --- | --- | --- |
|  |  | **1** | **2** | **3** | **4** | **5** | **6** | **7** | **8** | **9** | **10** | **11** | **12** | **13** | **14** | **15** | **16** | **17** | **18** | **19** | **20** |
| **Chelex®100** | RFLP | GG | GG | AA | GG | GG | GG | GG | GG | GG | GA | GG | GG | GA | GG | GA | GA | GG | GA | GG | GG |
|  | RT-PCR | GG | GG | AA | GG | GG | GG | GG | GG | GG | GA | GG | GG | GA | GG | GA | GA | GG | GA | GG | GG |
| **QIAamp DNA Mini Kit** | RFLP | GG | GG | AA | GG | GG | GG | GG | GG | GG | GA | GG | GG | GA | GG | GA | GA | GG | GA | GG | GG |
|  | RT-PCR | GG | GG | AA | GG | GG | GG | GG | GG | GG | GA | GG | GG | GA | GG | GA | GA | GG | GA | GG | GG |
| **Phenol-Chloroform** | RFLP | GG | GG | AA | GG | GG | GG | GG | GG | GG | GA | GG | GG | GA | GG | GA | GA | GG | GA | GG | GG |
|  | RT-PCR | GG | GG | AA | GG | GG | GG | GG | GG | GG | GA | GG | GG | GA | GG | GA | GA | GG | GA | GG | GG |
