# Supplementary figures and images for: Comparison of DNA extraction methods for COVID-19 host genetics studies
Source: PLoS One. 2023 Oct 30;18(10):e0287551. doi: 10.1371/journal.pone.0287551 (PMC10615309; doi:10.1371/journal.pone.0287551)

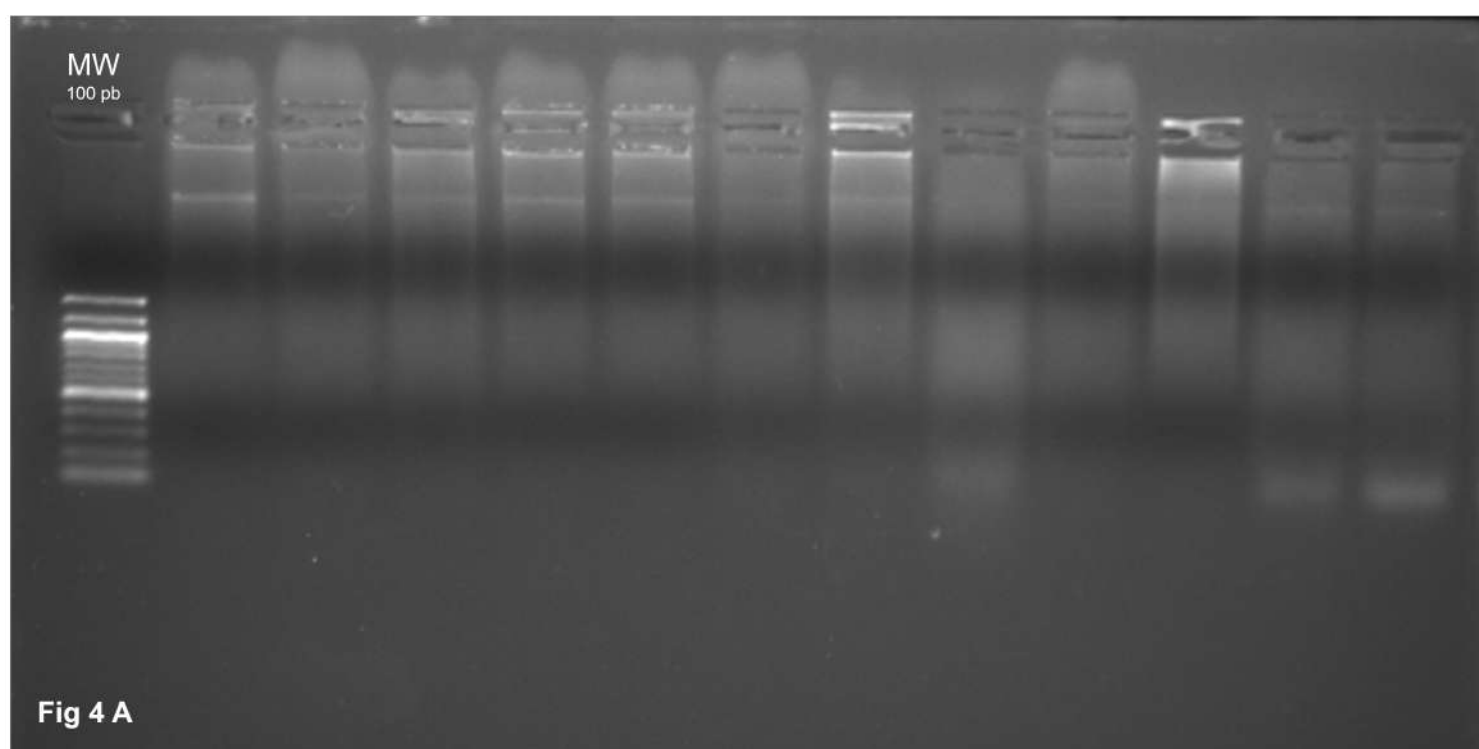

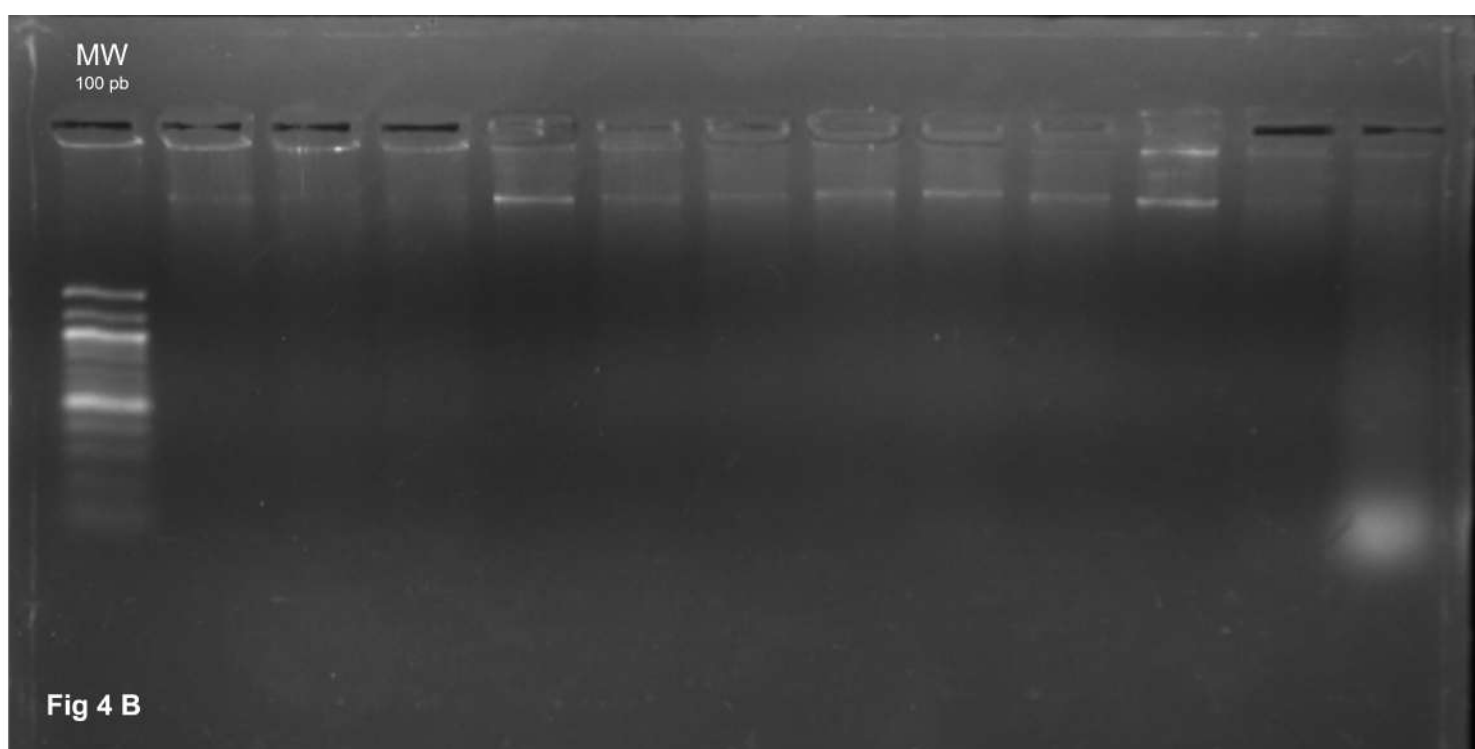

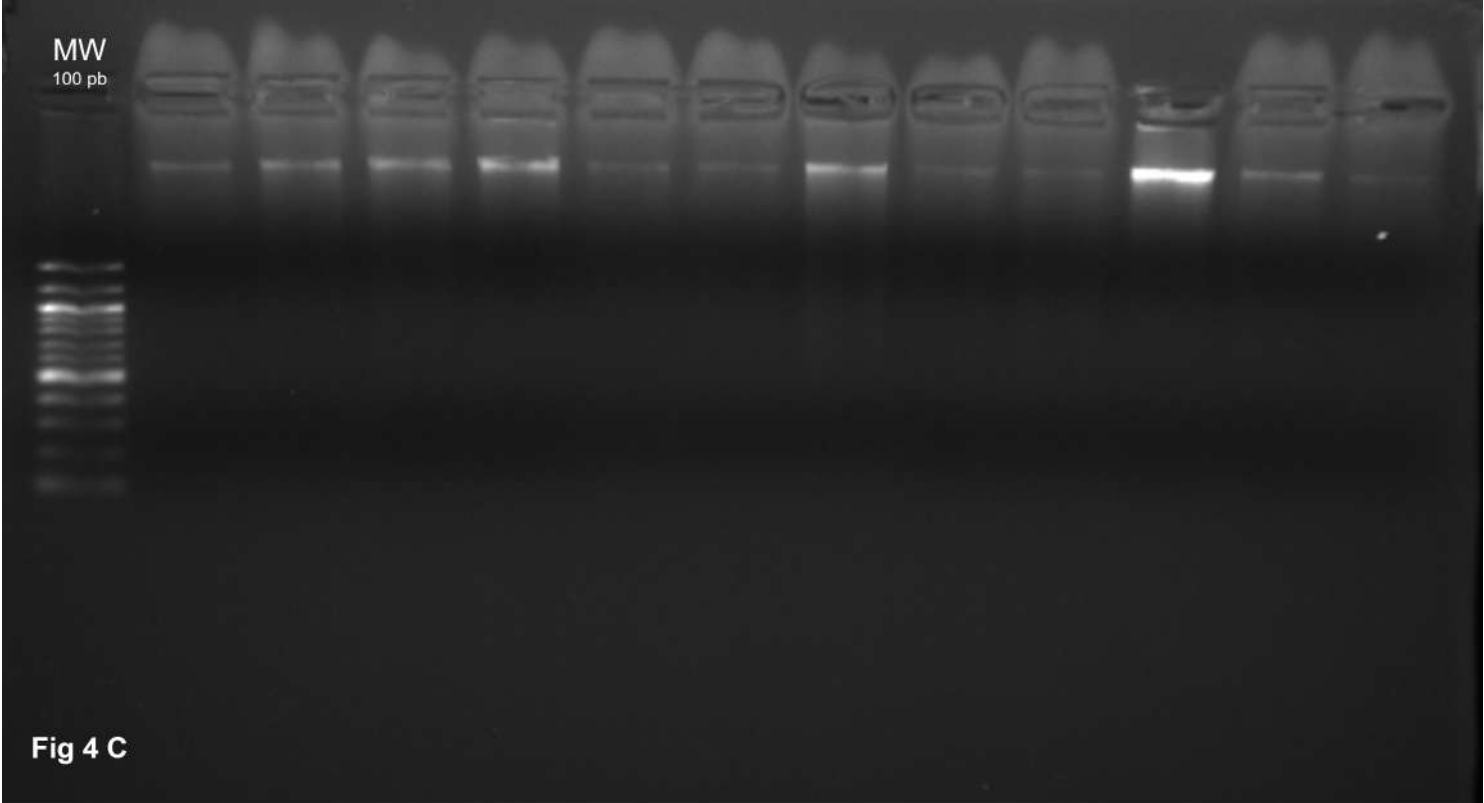

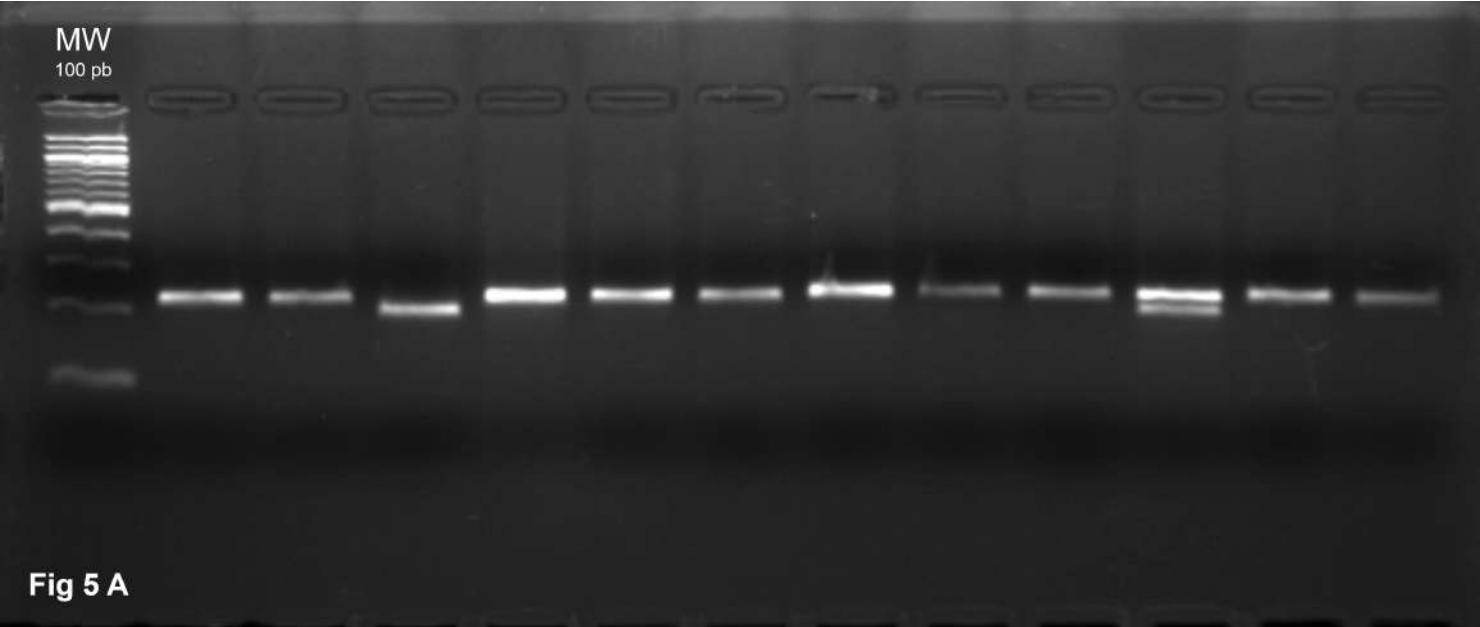

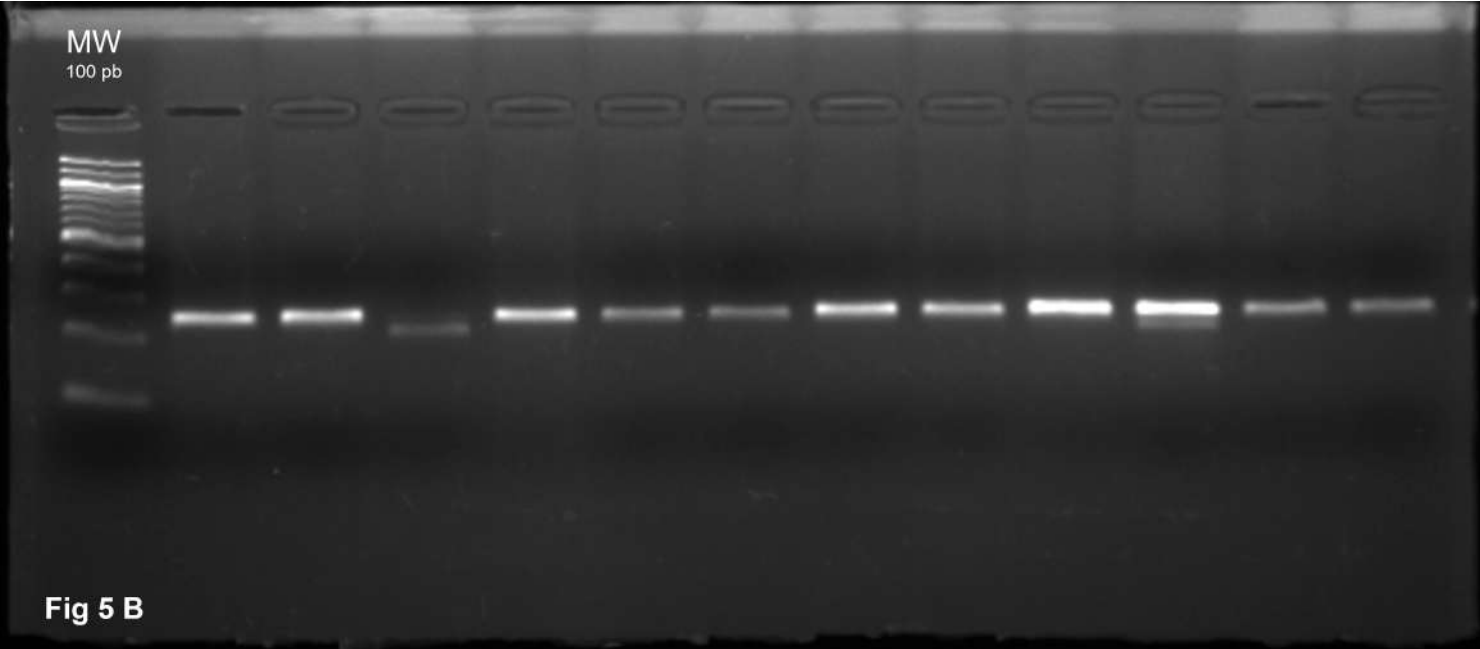

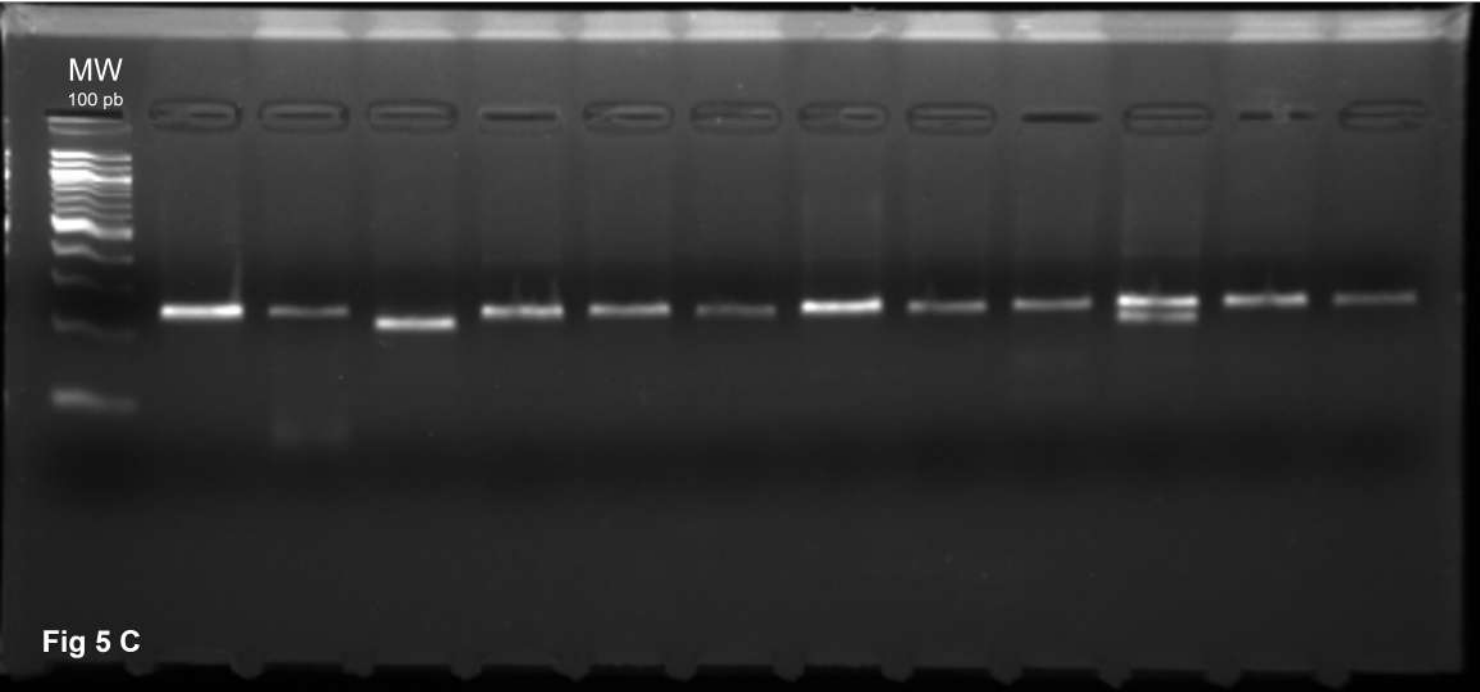

Fig 5 C

Supplement: S1 Raw images — (PDF) [file pone.0287551.s004.pdf]
